# Anacardic Acids from *Amphipterygium adstringens* Confer Cytoprotection against 5-Fluorouracil and Carboplatin Induced Blood Cell Toxicity While Increasing Antitumoral Activity and Survival in an Animal Model of Breast Cancer

**DOI:** 10.3390/molecules26113241

**Published:** 2021-05-28

**Authors:** Jairo Galot-Linaldi, Karla M. Hernández-Sánchez, Elizabet Estrada-Muñiz, Libia Vega

**Affiliations:** 1 Department of Toxicology, Center for Research and Advanced Studies of the National Polytechnic Institute, Ave. IPN 2508, San Pedro Zacatenco, GA Madero, Mexico City 07360, Mexico; jgalot@cinvestav.mx (J.G.-L.); eestrada@cinvestav.mx (E.E.-M.); 2 Escuela Nacional de Ciencias Biológicas del Instituto Politécnico Nacional, Prolongación de Carpio y Plan de Ayala s/n, Santo Tómas, Miguel Hidalgo, Mexico City 11340, Mexico; kmsanchez@gmail.com

**Keywords:** *Amphiterygium adstringens*, anacardic acids, myeloprotective, antineoplastic agent, autologous tumor model

## Abstract

*Amphipterygium adstringens* (cuachalalate) contains anacardic acids (AAs) such as 6-pentadecyl salicylic acid (6SA) that show immunomodulatory and antitumor activity with minimal or no secondary adverse effects. By contrast, most chemotherapeutic agents, such as 5-fluorouracil (5-FU) and carboplatin (CbPt), induce myelosuppression and leukopenia. Here, we investigated the myeloprotective and antineoplastic potential of an AA extract or the 6SA as monotherapy or in combination with commonly used chemotherapeutic agents (5-FU and CbPt) to determine the cytoprotective action of 6SA on immune cells. Treatment of Balb/c breast tumor-bearing female mice with an AA mixture or 6SA did not induce the myelosuppression or leukopenia observed with 5-FU and CbPt. The co-administration of AA mixture or isolated 6SA with 5-FU or CbPt reduced the apoptosis of circulating blood cells and bone marrow cells. Treatment of 4T1 breast tumor-bearing mice with the AA mixture or 6SA reduced tumor growth and lung metastasis and increased the survival rate compared with monotherapies. An increased effect was observed in tumor reduction with the combination of 6SA and CbPt. In conclusion, AAs have important myeloprotective and antineoplastic effects, and they can improve the efficiency of chemotherapeutics, thereby protecting the organism against the toxic effects of drugs such as 5-FU and CbPt.

## 1. Introduction

Cancer is a widespread human health problem and a leading cause of death worldwide [1]. The first choice for cancer treatment is chemotherapy, which is the use of drugs to destroy cancer cells. Chemotherapeutic drugs, such as 5-fluorouracil (5-FU) and carboplatin (CbPt), are commonly used to treat different types of cancer [2]; however, these drugs inevitably produce adverse side effects, such as myelosuppression and leukopenia [3]. Interestingly, the toxic effects of chemotherapy can be ameliorated by the administration of herbal plants or the natural compounds they contain. For example, curcumin, a natural polyphenol extracted from turmeric, attenuates CbPt-induced myelosuppression and increases the survival rate of tumor-bearing animals [4]. Similarly, treatment of rats with extracts from the lacquer tree (*Rhus verniciflua* Stoke) improves the resistance of rats to cisplatin chemotherapy-related adverse effects in the gastrointestinal tract and bone marrow [5].

**Figure 1:**
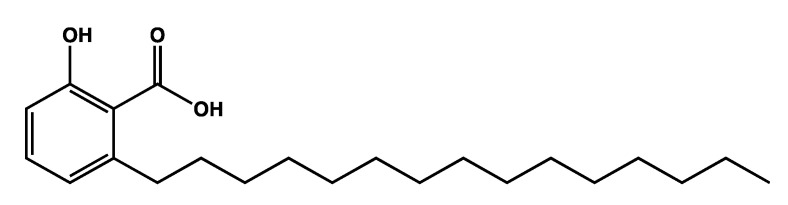
Chemical structure of 6-pentadecyl salicylic acid (6SA).

*Amphipterygium adstringens* (Schltdl.) Standl., of the Anacardiaceae family, known as “cuachalalate”, is a plant endemic to Mexico. The bark is used in traditional Mexican medicine to treat several diseases, mainly gastrointestinal disorders [6,7]. Phytochemical studies of *A. adstringens* bark have revealed many active compounds, such as triterpenes and flavonoids, as well as long-chain phenolic acids that are classified together as anacardic acids (AAs) [6,8,9]. The biological effects of AAs include cytotoxic, antibacterial, and antiproliferative activities [6,10,11,12,13,14]. The main AA found in the bark of *A. adstringens* is 6-pentadecyl salicylic acid (6SA) (Figure 1)*,* which is a well-characterized histone acetyltransferase inhibitor [15,16] that shows cytotoxic and antiproliferative activities in different cancer cell lines [12,13,14,15,16,17,18,19,20,21,22,23] and antineoplastic activity in xenografts in immunodeficient [19,24] and autologous cancer [25] models. 6SA also does not display cytotoxic effects in normal human peripheral blood mononuclear cells, in contrast to the most commonly used antineoplastic agents, which are cytotoxic to blood cells [17]. 6SA also has an important immunomodulatory effect, as it increases the migration and phagocytic activity of macrophages [26,27]. Supplementation with AAs such as 6SA can also have a potential protective role against oxidative and inflammatory disorders in the lungs [28].

6SA has known immunomodulatory activity, and it can mitigate the toxicity of the chemotherapy drug taxol (although it interferes with the drug’s antineoplastic activity) [25]. The aim of the present study was to evaluate the myeloprotective potential of AAs from *A. adstringens* and of commercially available 6SA, as well as their chemotherapeutic effects as monotherapy and in combination with the chemotherapy drugs 5-FU and CbPt in an immunocompetent autologous mouse model of stage IV breast cancer (metastatic cells).

## 2. Results

### 2.1. Analysis of AAs in a Hexane Extract from the Bark of A. adstringens

**Figure 2:**
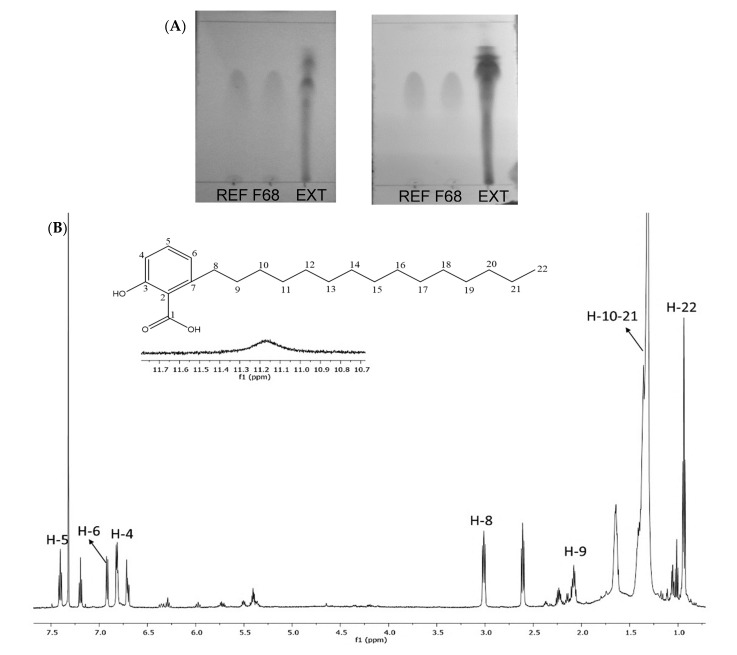
AA mixture characterization. Thin layer chromatography of 6SA (REF), fraction 68 of the AA mixture (F68), and the complete hexane extract from the bark of *A. adstringens* (EXT) by short wavelength ultraviolet light and cerium molybdate stain (**A**). 1H NMR spectrum (600 MHz, CDCl3) of fraction 68 (**B**): δ 7.25 (t, 1H, *J =* 7.25 Hz, H-5), 6.92 (dd, 1H, *J* = 7.62, 4.01 Hz, H-6), 6.48 (dd, 1H, *J* = 7.1, 3.8 Hz, H-4), 2.82 (m, 2H, H-8), 1.6 (m, 2H, H-9), 1.28 (m, 26H, H-10-21), 0.89 (t, 3H, *J* = 6.95, 6.95 Hz, H-22).

The hexane extract was separated by column chromatography (CC) and thin-layer chromatography (TLC) (Figure 2A). Subsequent ^1^H nuclear magnetic resonance and mass spectrometry analysis (Figure 2B and Supplementary Figure S1) indicated a substantial presence of 6SA in fraction 68 (Supplementary Materials: spectral data), as expected from the previous literature [6,9,11]. According to Mata et al. [9] and Castillo-Juárez et al. [6], the extraction method used here for the bark of *A. adstringens* produces an AA mixture containing 6SA (approximately 50%), 6-hexadecyl salicylic acid (6–7%), 6-heptadecyl salicylic acid (28–29%), 6-nonadecyl salicylic acid (7.5–8.5%), and monounsaturated 6-[15′(Z)-nonadecenyl]-salicylic (6.5–8.6%) [6,9]. Mass spectrometry analysis exhibited a pattern of peaks consistent with previously reported data in the literature for AA mixtures and 6SA from *A. adstringens* [6,9].

### 2.2. Myelotoxicity of Chemotherapeutic Agents and the Myeloprotective Effect of Anacardic Acids

We treated 4T1 breast cancer-bearing female Balb/c mice with therapeutic doses of 5-FU (40 mg/kg) and CbPt (100 mg/kg), or with an AA mixture (6 mg/kg) or commercial 6SA (6 mg/kg) alone for 21 days. The treatments with CbPt, and particularly 5-FU, dramatically decreased the total number of cells in the circulating blood and in the bone marrow (Table 1). Conversely, treatments with the AA mixture or 6SA did not change the total count of circulating blood cells or bone marrow cells. The administration of a combination of AAs and 5-FU co-treatment resulted in a marginal increase in the total cell numbers in the blood but had no effect on the loss of bone marrow cells. By contrast, the combination of 6SA and 5-FU significantly reduced the cell loss in the circulating blood and in bone marrow observed when 5-FU was administered alone, although the levels were still low when compared to the cell numbers in the control group. The combined treatment with 6SA and CbPt resulted in an increase in the circulating blood cell numbers when compared to the CbPt treatment alone, and the numbers did not differ from those of the control group, indicating that 6SA successfully protected circulating leukocytes from CbPt-induced cytotoxicity. We observed the same results when we used the combination of AA with CbPt (data not shown). Protection of the bone marrow cells was also evident, but the numbers did not reach the levels seen in the control group.

**Figure 3:**
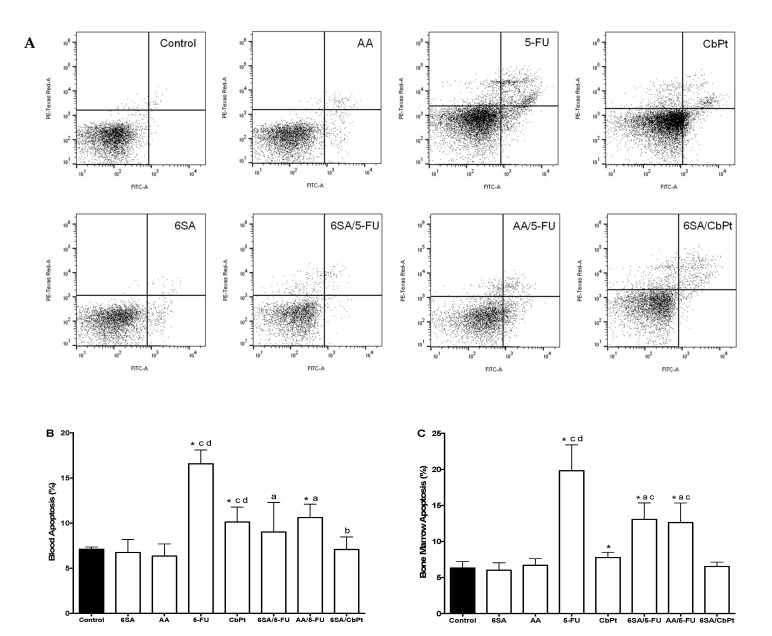
Anacardic acids mixture and 6SA decrease leukopenia and myelosuppression caused by 5-FU and CbPt. Mice were injected with 4T1 cells (5 × 10^3^ in 50 μL), and 8 days after tumor implantation, they received 6SA (6 mg/kg), AA (6 mg/kg), 5-FU (40 mg/kg), CbPt (100 mg/kg), 6SA/5-FU (6 + 40 mg/kg), AA/5-FU (6 + 40 mg/kg), or 6SA/CbPt (6 + 100 mg/kg) for 21 days. Flow cytometry of annexin V and propidium iodide stained (**A**) blood (**B**) or bone marrow cells (**C**). Mean ± SD, n = 6. * *p* < 0.05 One-Way ANOVA post hoc Bonferroni vs. control, ^a^ vs. 5-FU, ^b^ vs. CbPt, ^c^ vs. 6SA, or ^d^ vs. AA.

The reduction in the cell numbers was due to apoptosis, as shown by flow cytometry analysis of annexin V and evaluation of propidium iodide staining (Figure 3A). Treatments with 5-FU and CbPt increased apoptosis in blood cells (Figure 3B) and in bone marrow cells (Figure 3C), while AA or 6SA did not induce apoptosis in either cell type. Co-treatments with AA or 6SA and 5-FU significantly reduced apoptosis in blood cells (Figure 3B) and in bone marrow cells (Figure 3C), although the numbers of apoptotic cells were still higher than in the control group. The co-treatment with 6SA (or AA) and CbPt reduced the numbers of apoptotic cells observed with CbPt to the control group numbers, indicating that 6SA effectively protected blood cells and bone marrow cells from CbPt-induced apoptosis.

We examined whether the reduction in the blood cell numbers was due to a decrease in a particular subpopulation of leukocytes by performing differential leukocyte counts in blood samples from the animals. Table 2 shows that the treatments with the AA mixture and 6SA did not reduce the numbers of most cell types. In fact, these treatments increased the numbers of band lymphocytes and monocytes, as previously reported [19]. By contrast, treatment with 5-FU and CbPt decreased the numbers of neutrophils, basophils, and band lymphocytes (5-FU), while they increased the number of immature circulating cells, lymphocytes (5-FU), or band lymphocytes (CbPt). The co-administration of 5-FU with either the AA mixture or 6SA resulted in an increase in the number of monocytes above the numbers seen in the control group. A recovery from the cytotoxic effects of 5-FU was observed in the number of neutrophils, band lymphocytes, and basophils and a reduction in the number of immature circulating cells. The number of lymphocytes also increased above the control group numbers in response to the combined treatments with AA or 6SA and 5-FU. Co-administration of 6SA or AA (data not shown) and CbPt increased the numbers of neutrophils, lymphocytes, and monocytes and reduced the numbers of immature cells.

This protective effect of the AA mixture and 6SA against the toxicity of 5-FU and CbPt correlates with the effects observed in the apoptosis induction and could be related to the immunomodulatory effect of 6SA that we have previously reported [20,21].

### 2.3. Effect of Treatments on Animal Overall Systemic Health

In addition to evaluating the cellular parameters of leukopenia and myelosuppression, we also considered a gross parameter of systemic toxicity commonly used in cancer treatments. The induction of the tumor model causes an increase in the size of the spleen and lungs, as previously reported [29,30] (Table 3. Naïve vs. control). After three weeks of treatment, no significant difference was noted in body weight gain after the treatments with 6SA, AA, 6SA/CbPt, and AA/CbPt (data not shown), while the treatment with 5-FU, CbPt, and 5-FU/6SA caused a significant decrease in body weight gain (Table 3). All treatments decreased the relative weight of the lungs to a normal value; this event is closely related to the reduction of the metastatic foci in this organ. No significant changes were observed in the liver and kidney relative weights with monotherapies, but a significant increase in these organs occurred with the co-treatments. Most treatments reduced the splenomegaly caused by the presence of the tumor except for 6SA and 5-FU (Table 3). These data indicate that AA and 6SA treatments reduced the systemic toxicity compared to 5-FU and CbPt treatments and that the co-treatments reduced the toxicity of the classical chemotherapeutic agents.

### 2.4. In Vivo Antitumoral, Antimetastatic, and Survival Effects of 6SA, Chemotherapeutics, and Co-Treatments

**Figure 4:**
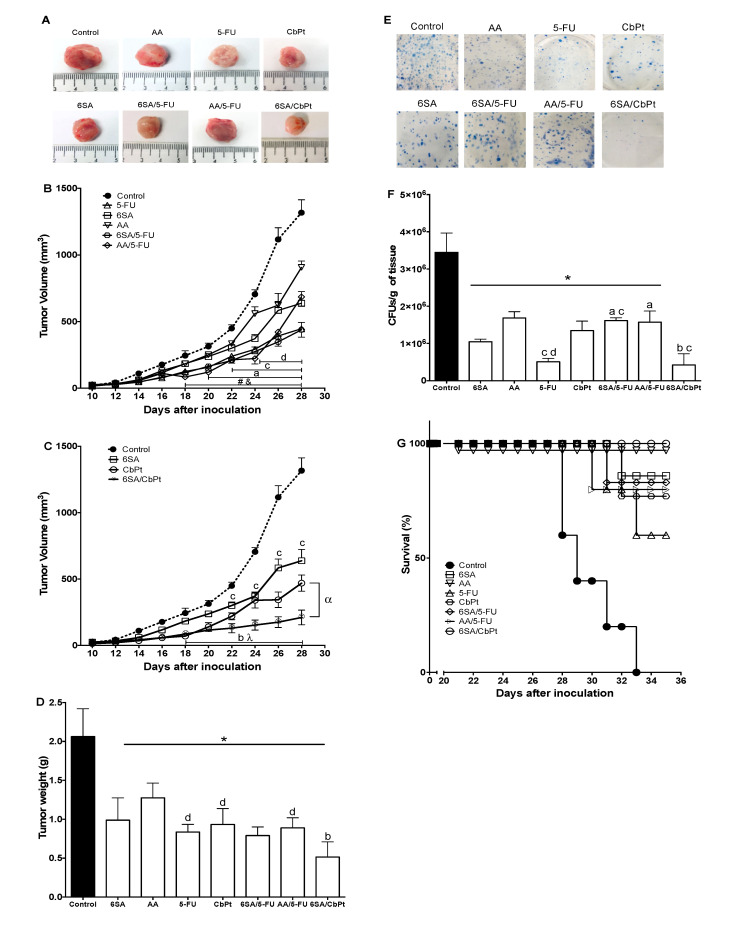
Anacardic acids inhibit tumor growth, decrease lung metastasis, and increase animal survival. Mice were injected with 4T1 cells (5 × 10^3^ in 50 μL), and 8 days after tumor implantation, they received 6SA (6 mg/kg), AA (6 mg/kg), 5-FU (40 mg/kg), CbPt (100 mg/kg), 6SA/5-FU (6 + 40 mg/kg), AA/5-FU (6 + 40 mg/kg), or 6SA/CbPt (6 + 100 mg/kg) for 21 days. Excised tumors after 21 days of treatments (**A**), tumor volume (**B**,**C**), and tumor weight (**D**). Metastatic assay of 4T1 cells (**E**) and the percentage of 4T1 colony cells in lung tissue (**F**). Survival rate of animals after treatments (**G**). Mean ± SEM (**B**,**C**), Mean ± SD (**D**,**F**), n = 6. ^a^ 5-FU, ^b^ CbPt, ^c^ 6SA, ^d^ AA, ^#^ 6SA/5-FU, ^&^ AA/5-FU, and ^λ^ 6SA/CbPt *p* < 0.05 Two-Way ANOVA post hoc Bonferroni vs. control, * *p* < 0.05 One-Way ANOVA post hoc Bonferroni vs. control, ^a^ vs. 5-FU, ^b^ vs. CbPt, and ^d^ vs AA. ^α^ *p* < 0.05 Two-Way ANOVA post hoc Bonferroni CbPt vs. 6SA/CbPt.

Since the final desired outcome of the therapeutic treatments is the reduction of primary and metastatic tumors, we evaluated the effect of these treatments on tumor development (Figure 4A). All the monotherapies administered to female Balb/c tumor-bearing mice significantly reduced the tumor volume (Figure 4B,C) and the tumor weight (Figure 4D) when compared to the control group (untreated) after 21 days. The antitumor effect of 6SA and AA (with an estimated dose of 3 mg/kg of 6SA) significantly reduced the tumor volume by 52 and 31%, respectively, although 5-FU (66%) and CbPt (65%) decreased the tumor volume more effectively than AA or 6SA alone (Figure 4B,C). The combination treatments with 5-FU and either AA or 6SA decreased the tumor volume and weight in a similar proportion to 5-FU alone (Figure 4B,D). Conversely, the combined treatment of CbPt and 6SA significantly decreased the tumor volume and weight (Figure 4C,D) compared to the monotherapies, indicating an increase in the effect of 6SA and CbPt alone. The effects observed with the treatment of AA and CbPt were the same as those observed using 6SA and CbPt (data not shown).

One major problem in cancer treatment is obtaining an effective reduction of the migration of tumor cells to other organs (metastasis). The breast cancer tumor model used here allowed us to identify migrating tumor cells from the primary tumor as they can grow in an environment containing 6-TG (where all normal cells die) (Figure 4E). Using this approach, we evaluated the migration of 4T1 cells toward the lungs and liver after three weeks of treatment. No metastatic foci were found in the liver at that time, which was probably because of the small number of 4T1 cells that were inoculated and the time of evaluation [29,30]. All monotherapy treatments significantly reduced metastasis to the lung compared to the control group, although 5-FU was the most effective and AA only reduced metastasis by 50%. The combined treatments with 5-FU and either AA or 6SA had no better effect than that obtained with 5-FU alone. On the contrary, the co-treatment only reduced metastasis by 50%, similar to AA alone, indicating an interference in this particular effect of 5-FU by 6SA (Figure 4F). In comparison, CbPt alone was not very effective in reducing the metastasis of 4T1 cells to the lungs, but when used in combination with 6SA (or AA, data not shown), the co-treatment significantly decreased the migration of 4T1 cells to the lung compared to the control (Figure 4F). In fact, this combination was as effective as 5-FU alone in reducing lung metastasis.

The goal of chemotherapy is to reduce tumor growth and to prolong the life of the patient with sufficient quality. Therefore, we evaluated the survival rate of the animals given the different treatments. We registered the spontaneous deaths of the animals or sacrificed them when the tumor reached 2000 mm^3^ in volume or presented an inconvenience to the mobility of the animal, as established in preclinical protocols [31]. Mice in the control group died or were sacrificed between 28 and 32 days after initiating the treatments (only vehicle in this group) (Figure 4G). Treatment with 5-FU increased the survival of the animals up to 60% after 36 days (when the tumors reached 2 cm^3^), while CbPt, 6SA, and 6SA/5-FU increased survival up to 80% after 36 days. Treatment with AA and the co-treatment with 6SA and CbPt increased survival of the animals up to 100% (as the treatment with AA and CbPt, data not shown) before the tumor reached the maximal permitted size (Figure 4G). In addition to these data, we also observed changes in the animal’s behavior, appearance, and activities (such as grooming) that were consistent with the survival rate and the weight loss (Table 3) (Data not shown).

## 3. Discussion

The bark of *A. adstringens* has been used in Mexican traditional medicine mainly to treat gastrointestinal diseases that range from gastritis to gastric cancer. Some reports indicate that the main biologically active compounds in *A. adstringens* are the AAs and that 6SA is the component that displays antimicrobial and antineoplastic effects [6,11,12,13,14,19,24]. We isolated and identified a mixture of AAs with a high proportion of 6SA from a hexane extract of the bark of *A. adstringens* with the same characteristics as other groups have reported [6,9,12].

In this study, we demonstrated that the AA mixture and commercially available 6SA have myeloprotective effects in a chemotherapy regimen based on 5-FU or CbPt. These commonly used antineoplastics significantly increase blood and bone marrow cell apoptosis and circulation of immature cells in the blood, thereby causing myelosuppression and leukopenia in human patients. The co-treatment with the AA mixture or 6SA improved these cellular parameters in the individual mice. Several studies have demonstrated the myeloprotective effect of some plant extracts or their active metabolites in chemotherapy. For example, the aqueous extract of *R. verniciflua* (also from the Anacardiaceae family, such as *A. adstringens*) improves the resistance of rats to the adverse effects of chemotherapy in the gastrointestinal tract and the bone marrow [5]. Similarly, curcumin, a natural polyphenol, increases the tolerance of bone marrow cells to the toxic effects produced by chemotherapy and suppresses the defective hematopoiesis induced by tumor-derived VEGF in a tumor model [4].

These effects have been linked to the capacity of these compounds to inhibit acetyl-transferases [16,18,32,33], which is a mechanism that is well characterized for 6SA. The myeloprotective effect and the protection of WBCs could be related to the previous immunomodulatory effect described for 6SA by our group and others [13,25], namely the ability of 6SA to induce the phosphorylation of kinases important for the proliferation of immune cells, particularly T lymphocytes and differentiated macrophages. Thus, this mechanism of action could increase the resistance of immune cells to the damage and apoptotic signals of chemotherapeutic agents such as taxol, 5-FU, or CbPt.

In our study, the AA mixture and 6SA produced a strong antitumor effect by reducing the volume and weight of the tumor. The volume and tumor weight were also decreased in a previous study after 6SA administration (2 mg/kg daily for 30 days) in mice bearing human prostate tumor xenografts [18]. The suppression of tumor growth by 6SA (2 mg/kg daily for 14 days) has also been reported in mice with HepG2 tumor xenografts [19]. Gnanaprakasam et al. [25] used a syngeneic murine model similar to the one used in the present study and showed that 6SA reduced tumor volume and size by inducing caspase-8-mediated apoptosis, and it reduced lung metastasis. The antitumor effects produced by 6SA have been related to a decrease in the expression of proteins involved in cell proliferation and survival, such as Src [24], and to the induction of endoplasmic reticulum stress by 6SA [19]. Similarly, the ability of 6SA to inhibit histone acetyltransferases may have an important role in the reported biological effects [15,18,34]. In the present study, 6SA showed high antineoplastic efficiency when combined with CbPt, but not with 5-FU. A large number of studies have shown the potential of using natural products such as curcumin and resveratrol in cancer treatment, as well as their synergistic effects with chemotherapeutic agents [35,36,37].

Metastasis refers to the spread of cancer cells from the primary tumor to surrounding tissues and distant organs and is the leading cause of cancer morbidity and mortality. Metastasis is estimated to be responsible for approximately 90% of cancer deaths [38]. In our experimental model, in comparison to other previously reported studies, we observed that all treatments decreased lung metastasis of 4T1 cells, but that the combined treatment of 6SA with CbPt was more effective than the combination of 6SA and 5-FU in reducing lung metastasis of 4T1 cells (triple negative breast cancer stage IV). We believe that the relative “unresponsiveness” and lack of an increased or summatory effect of 6SA on 5-FU treatment is related to the ability of 5-FU to alter the molecular targets of 6SA, as 5-FU can induce the degradation of p300/CBP and decrease PCAF expression [39,40], which are particular targets of 6SA.

Overall, the immune cell cytoprotective effects and antitumoral activity of 6SA increased the survival rate and the quality of life of the animals, indicating that these treatments could be beneficial to human cancer patients.

## 4. Materials and Methods

### 4.1. Plant Material and Reagents

We obtained the bark of *A. adstringens* from a registered provider (Pacalli S. de R.L. de C.V. Monterrey, Nuevo Leon, Mexico; Batch number CCO001011117). Its correct scientific name was verified in http://www.theplantlist.org (accessed on 10th February 2017) (kew-2634734). We obtained carboplatin (CbPt, M.W. 371.249 g/mol), veterinary isoflurane, and heparin (1000 UL/mL) from PiSa Laboratories (Mexico City, Mexico). We purchased 5-FU (M.W. 130.07 g/mol, 99% purity, Cat. F6627), 6-thioguanine (6-TG, M.W. 167.19 g/mol, 98% purity, Cat. A4882), and trypan blue (Cat. T8154) from Sigma-Aldrich (St. Louis, MO, USA). We obtained the 6SA (M.W. 348.5 g/mol, 97.1% purity) from Calbiochem (San Diego, CA, USA) (Batch number 2997603). Collagenase A was obtained from Roche (Penzberg, Germany). We purchased all other reagents from Sigma-Aldrich, J.T. Baker (Deventer, Holland), and Gibco (Grand Island, NY, USA), as indicated.

### 4.2. Extraction and Isolation of the AA Mixture from A. adstringens

We extracted finely ground bark of *A. adstringens* (200 g) in hexane (Química Rique S.A. de C.V.) at room temperature over one week. Then, we concentrated the extract under reduced pressure using a rotary evaporator. This yielded 3.5 g of a dark green oily residue, which represented 1.75% (*w*/*w*) of *A. adstringens* bark. The extract was stored at 4 °C in dark conditions until use.

We analyzed the composition of the extract by thin layer chromatography (TLC, precoated sheets of silica gel, Merck 60F-254) using the Hex:CH_2_Cl_2_:EtOAc (20:20:10) system as the developing solvent, followed by visualization under UV light or by spraying with cerium molybdate or cerium sulfate.

For further purification, we loaded 2.7 g of the hexane extract onto a chromatography column (silica gel, Merck 230–400 mesh) and used the same developing solvent to fractionate the extract. Then, we analyzed the fractions by comparing their ^1^H NMR spectra (Varian NMR System at 600 MHz using deuterated chloroform (CDCl3) and deuterated methanol (Methanol-d4) as solvents (or a combination), followed by mass spectrometry analysis (Bruker AmaZon Speed spectrometer by electrospray ionization) using published data and TLC comparisons, where 6SA (6-pentadecyl salicylic acid, C15:0) was used as a reference. Fractions 35 to 68 yielded a yellowish white solid (180 mg); the analysis indicated a mixture of long-chain phenolic acids, which are known as AAs. Fraction 68 (AA mixture) was selected due to its high 6SA content (Figure 1).

According to Mata et al. [9] and other reports [6], a kilogram of *A. adstringens* bark contains 0.960 g of AA mixture, with 0.480 g corresponding to 6SA (approximately 50%).

### 4.3. Animals and Ethics

We used female Balb/c mice (6–8 weeks old) from the CINVESTAV animal facility. The animals were housed in a ventilated cage (Super mouse model 750) in an OC 2100 model rack (Lab Products Inc., Seaford, DE, USA) and kept under a double full barrier in a clean air-conditioned room with double HEPA air filtration (99.99% efficiency and 0.3 microns), controlled temperature between 20 and 24 °C, relative humidity between 40 and 70%, and a 12:12 day/night control cycle, with noise below 70 db, 0 ppm NH_3_ in the microenvironment at day 7 of bedding change, and vibrations below 0.05 g. All micro isolator cage bedding was autoclaved, and the animals had free access to food (PicoLab Rodent Diet 5058, sterilized by radiation) and to ultrafiltered tap water generated by Hydropac 2500 equipment (Lab Products Inc., Seaford, DE, USA). Animals were acclimated for 1 week prior to experimentation and were sacrificed by isoflurane overdose. All procedures followed the Mexican Guideline Regulations of Animal Care and Maintenance (NOM-062-ZOO-1999, 2001), (https://www.fmvz.unam.mx/fmvz/principal/archivos/062ZOO.PDF (accessed on 15 July 2015). The Internal Committee for the Care and Use of Laboratory Animals (CICUAL) of CINVESTAV approved the experimental protocol (CICUAL, Protocol 0082–14).

### 4.4. 4T1 Cell Line

We obtained the highly metastatic 4T1-Balb/c cell line of breast adenocarcinoma for the mouse tumor model from the American Type Culture Collection (ATCC-CRL-2539, Manassas, VA, USA). Cells were cultured in RPMI-1640 medium supplemented with penicillin (10 U/mL)/streptomycin (10 μg/mL), non-essential amino acids (1 mM) (Sigma-Aldrich), and 10% heat-inactivated fetal bovine serum (FBS; Gibco) at 37 °C in a humidified atmosphere with 5% CO_2_. We used 4T1 cells from subculture pass 3 at no more than 80% confluence to inoculate animals.

On the day of inoculation, subconfluent 4T1 cell cultures were harvested with 0.25% trypsin-0.53 mM EDTA solution and resuspended in phosphate-buffered saline (PBS). We determined the cell number and viability (>90%) using the trypan blue exclusion assay.

### 4.5. Experimental Protocol

#### 4.5.1. Tumor Development and Treatments

We inoculated Balb/c female mice as described previously [25] by subcutaneous (s.c.) injection of 5 × 10^3^ 4T1 cells in 50 μL of PBS into the inguinal mammary fat pad using a 27G needle. We monitored the tumor onset by palpating the injection area with the index finger and thumb for the presence of a tumor. Eight days after 4T1 cell implantation, when mice showed palpable tumors, we randomly divided the animals into corresponding treatment groups (6 animals per group). We applied the treatments intravenously (i.v.) via the tail vein once a week for 3 weeks. The treatments were (1) AAs; 6 mg/kg, (2) 6SA; 6 mg/kg [24,25], (3) 5-FU; 40 mg/kg [41], (4) CbPt; 100 mg/kg [42], (5) AA/5-FU; 6 + 40 mg/kg, (6) 6SA/5-FU; 6 + 40 mg/kg, (7) 6SA/CbPt; 6 + 100 mg/kg or, (8) vehicle (50 µL of PBS:DMSO:Tween-20; 7.6:2:0.4). A group treated with AA/CbPt (6 + 100 mg/kg) was performed, but as those results were the same as the group treated with 6SA and CbPt, we did not included those data in the tables.

#### 4.5.2. Tumor Growth

We assessed the pharmacological effects of treatments on tumor growth by measuring the tumor size every two days during the treatment period using a precision caliper. We calculated the volume using the formula V = 0.5 × C^2^ × L, where V is the volume (mm^3^), C is the short diameter, and L is the long diameter of the tumor [29].

After completion of the treatment period, we sacrificed the animals with an inhaled isoflurane overdose. We collected the blood by cardiac puncture with a heparinized syringe to register the total and the differential WBC count and to determine apoptosis. We performed a complete postmortem examination on each animal. Specifically, we dissected the liver, kidneys, spleen, lungs, and the tumor from the body to register the absolute and relative organ weight (ROW) using the formula ROW = absolute organ weight (g)/body weight (100).

#### 4.5.3. Determination of Lung Metastasis by Clonogenic Assay

We investigated the effect of the different treatments on the lung metastasis of 4T1 cells using a clonogenic assay according to previous reports [29,30], with minor modifications. Briefly, we used 0.2 g of lung tissue placed on a culture plate containing PBS (500 μL) and chopped the tissue into small pieces (<1 mm^3^) with a sterile blade. Subsequently, we added 300 μL of collagenase A (2.5 mg/mL in PBS/KCl 1 M) and incubated the tissue for 30 min at 37 °C with frequent agitation. After incubation, we filtered the cell suspension through a 40 μm cell strainer and recovered all cells into Hank’s Balanced Salt Solution (HBSS, 5 mL). Finally, we resuspended one aliquot (5 μL) in 5 mL RPMI 1640 supplemented with 10% FBS, 1% penicillin-streptomycin, and 60 µM 6-TG on a culture plate (60 mm). We incubated the cultures at 37 °C in a 5% CO_2_ atmosphere and kept them under observation for 8 days, which was sufficient time to allow 4T1 cell colony formation. On day 8, we discarded the medium from the plates and fixed the cells with 100% methanol (5 min), stained them with methylene blue (0.03% in distillated water) for 5 min, and manually counted the number of colony formation units (CFUs) in the plate. We photographed the plates with a Leica EZ4 HD stereoscope microscope (8× magnification).

#### 4.5.4. Bone Marrow Collection

Both femora were dissected from each mouse, the skin and muscle tissue were removed, and both ends of the bone tips were severed with bone snips. We gently flushed the marrow from the channel into a microcentrifuge tube using a 25G needle with FBS (500 μL/femur or until bone turned white) to maximize the number of cells harvested. We assessed cell number by the 0.4% trypan blue exclusion assay and determined cell death by an apoptosis/necrosis assay, as described previously [25].

#### 4.5.5. Total and Differential White Blood Cell (WBC) Counts in Peripheral Blood

After the extraction of blood by cardiac puncture, we evaluated the total number of cells per ml using the trypan blue assay. Then, we prepared glass slides to determine the total and differential WBC count by staining with Giemsa-Wright stain (WG16-500 mL, Sigma-Aldrich) and determined the numbers of the different granulocyte series, lymphocytes, and monocytes in 100 leucocytes per sample using an Olympus CH30RF100 optical microscope (Olympus Optical Co., Ltd., Hatagaya, Shibuya-Ku, Tokyo, Japan) at 100× magnification as described elsewhere [43].

#### 4.5.6. Evaluation of Apoptosis in Leukocytes of Peripheral Blood and Bone Marrow Cells

Apoptosis was evaluated using a staining kit for the differential quantification of apoptosis and necrosis (FITC Annexin V Apoptosis Detection Kit II BD Biosciences 556570). One million cells of peripheral blood or bone marrow were transferred to a 5 mL culture tube, washed twice with cold PBS, and then resuspended in 50 μL of 1× Binding Buffer (10 µL FITC Annexin V and 10 µL propidium iodide per mL). The cell suspensions were gently mixed and incubated for 5 min at RT (25 °C) in the dark, followed by the addition of 300 µL of PBS to each tube and immediate analysis by flow cytometry (BD LSRFORTESSA SORP, Becton Dickinson, Franklin Lakes, NJ, USA). The proportion of necrotic (red) and apoptotic (green and red/green) cells was evaluated in 50,000 events using Flow Jo software, as described previously [25].

#### 4.5.7. Survival Analysis

We determined whether the treatments modified the survival rate of 4T1 tumor-bearing mice by subjecting the animals (5 per group) with the different treatments described in Section 4.5.1 for 3 weeks and suspended the treatments. Mice were checked daily, and the day each died was recorded. When the tumor of any animal reached a size of 2 cm^3^, we sacrificed that animal for humanitarian reasons. We did not need to sacrifice animals earlier than that point due to severe weight loss or signs or symptoms of suffering or discomfort.

### 4.6. Statistical Analysis

We presented the results as the mean ± standard error (SE) or standard deviation (SD). We assessed the statistical significance of the data using two-way and one-way ANOVA, followed by a Bonferroni post hoc analysis or unpaired Student’s *t* test for comparison between two groups. We considered *p* < 0.05 to be statistically significant. All statistical analyses were performed using the GraphPad PRISM program 6.0.

## 5. Conclusions

The present study demonstrated that anacardic acids can reduce the toxic effects of chemotherapeutic agents such as 5-FU and CbPt on bone marrow and leukocytes, as reflected at the hematological and systemic levels. These effects may be a consequence of the immunomodulatory effect of 6SA, independent of its antineoplastic activity. 6SA can potentially be used in combination with other antineoplastic agents, such as CbPt, to induce an increased effect and achieve higher tumor reduction ratios in patients, thereby increasing their survival rate and quality of life. These findings suggest that anacardic acids from *A. adstringens*, and specifically 6SA, may serve as therapeutic agents for the treatment of breast cancer, particularly in hormone-unresponsive tumors that tend to be more difficult to treat, such as triple-negative 4T1 tumors.

Although the present results are promising, more research is needed to provide evidence for the increased effects of 6SA with other chemotherapeutic regimens and in other tumor types.

## Figures and Tables

**Table 1:** Blood and bone marrow cellularity of animals.

Tissue	Control	6SA	AA	5-FU	CbPt	6SA/5-FU	AA/5-FU	6SA/CbPt
Blood cel/mL (10^6^)	7.7 ± 1.1	6.1 ± 2.0	7.9 ± 1.4	3.0 ± 1.0 *^,c,d^	5.4 ± 0.7 *^,c,d^	5.0 ± 1.4 *^,a,c^	3.9 ± 1.1 *^,d^	7.0 ± 1.0 *^,b,c^
Bone Marrow cel/mL (10^6^)	2.3 ± 0.3	1.8 ± 0.2	1.7 ± 0.2	0.6 ± 0.2 *^,c,d^	1.0 ± 0.8 *^,c,d^	1.1 ± 0.7 *^,a c^	0.8 ± 0.2 *^,d^	1.4 ± 0.0 *^,b^

All animals bare 4T1 cells breast tumor. Mice were treated with 6SA (6 mg/kg), 5-FU (40 mg/kg), AA (6 mg/kg), CbPt (100 mg/kg), 6SA/5-FU (6 + 40 mg/kg), AA/5-FU (6 + 40 mg/kg), and 6SA/CbPt (6 + 100 mg/kg) for 21 days. Mean ± SD (n = 6). * *p* < 0.05 by One-Way ANOVA followed by Bonferroni test vs. control group, ^a^ vs. 5-FU, ^b^ vs. CbPt, ^c^ vs. 6SA and ^d^ vs. AA.

**Table 2:** WBC differential counts of animals.

WBC Type	Control	6SA	AA	5-FU	CbPt	6SA/5-FU	AA/5-FU	6SA/CbPt
Neutrophil	43.3 ± 0.9	36.8 ± 3.0	39.3 ± 3.1	16.0 ± 4.1 *^,c,d^	21.8 ± 8.2 *^,c,d^	34.6 ± 9.0 *^,a^	36.8 ± 1.5 ^a^	26.0 ± 3.6 *^,c^
Band	20.5 ± 1.7	27.5 ± 3.1 *	28.3 ± 4.0 *	12.0 ± 3.6 *^,c,d^	28.5 ± 4.7 *^,a^	19.3 ± 4.2 ^c^	22.8 ± 2.6 ^a^	14.6 ± 1.5 *^,b,c^
Lymphocyte	14.8 ± 2.1	13.5 ± 5.1 *	12.3 ± 1.9 *	24.3 ± 2.1 *^,c,d^	15.0 ± 4.4 ^a^	24.0 ± 7.2 *^,c^	23.7 ± 1.7 *^,d^	47.0 ± 5.6 *^,b,c^
Monocyte	3.5 ± 0.6	8.5 ± 0.6 *	6.0 ± 1.8 *	4.0 ± 0.8 ^c,d^	4.3 ± 1.7	8.0 ± 2.0 *^,a^	8.5 ± 2.6 *^,a^	6.3 ± 1.5 *
Basophil	0.5 ± 1.0	1.8 ± 1.3	0.8 ± 1.3	0.0 ± 0.0 ^c^	1.3 ± 0.9	1.3 ± 1.1 ^a^	0.0 ± 0.0	0.0 ± 0.0 ^c^
Eosinophil	1.8 ± 1.3	1.8 ± 1.3	0.0 ± 0.0 *	0.8 ± 0.5 ^d^	1.0 ± 0.8 ^d^	1.3 ± 1.1	0.3 ± 0.5	1.0 ± 0.0
Immature	14.5 ± 0.6	10.3 ± 4.5	14.0 ± 2.0	43.3 ± 5.9 *^,c,d^	28.5 ± 4.7 *^,c,d^	11.3 ± 3.1 ^a^	8.0 ± 1.4 *^,a,d^	5.0 ± 1.0 *^,b^

All animals bore 4T1 cell breast tumors. Mice were treated with 6SA (6 mg/kg), 5-FU (40 mg/kg), AA (6 mg/kg), CbPt (100 mg/kg), 6SA/5-FU (6 + 40 mg/kg), AA/5-FU (6 + 40 mg/kg) and 6SA/CbPt (6 + 100 mg/kg) for 21 days. Mean ± SD (n = 6). * *p* < 0.05 by two-way ANOVA followed by Bonferroni test vs. control group, ^a^ vs. 5-FU, ^b^ vs. CbPt, ^c^ vs. 6SA and ^d^ vs. AA.

**Table 3:** Relative weight (index) of different organs (ROW) and body weight gain of animals.

Organ	Naive	Control	6SA	AA	5-FU	CbPt	6SA/5-FU	AA/5-FU	6SA/CbPt
Spleen ^&^	0.3 ± 0.0	2.7 ± 0.3	2.6 ± 0.1	2.0 ± 1.0 *	2.7 ± 0.4	1.3 ± 0.1 *^,c^	1.1 ± 0.1 *^,a,c^	1.4 ± 0.5 *^,a^	1.7 ± 0.1 *^,c^
Lung	0.8 ± 0.0	2.5 ± 0.3	1.0 ± 0.1 *	1.3 ± 0.2 *	1.0 ± 1.1 *	1.1 ± 0.1 *	1.1 ± 0.2 *	1.1 ± 0.3 *	1.0 ± 0.1 *
Liver ^&^	5.1 ± 0.1	5.7 ± 0.1	5.3 ± 0.3	5.6 ± 0.6	6.1 ± 0.4	5.6 ± 0.5	6.4 ± 0.4 *	6.9 ± 0.8 *^,d^	7.2 ± 0.4 *^,b,c^
Kidney	1.1 ± 0.0	1.2 ± 0.0	1.2 ± 0.1	1.2 ± 0.1	1.2 ± 0.1	1.3 ± 0.4	1.3 ± 0.1	1.4 ± 0.1	1.5 ± 0.1 *^,c^
Body weight gain	1.9 ± 0.9	1.7 ± 1.0	1.18 ± 0.5	1.03 ± 0.5	−1.2 ± 0.7 *^,c,d^	−1.1 ± 0.7 *	−0.80 ± 0.3 *^,c^	−0.73 ± 0.7 *^,d^	0.63 ± 0.4 ^b^

Naïve animals did not have tumors, the rest of the animals had 4T1 cells breast tumors. Mice were treated with 6SA (6 mg/kg), 5-FU (40 mg/kg), AA (6 mg/kg), CbPt (100 mg/kg), 6SA/5-FU (6 + 40 mg/kg), AA/5-FU (6 + 40 mg/kg), and 6SA/CbPt (6 + 100 mg/kg) for 21 days. ^&^ Relative organ weight (ROW) was determined using the formula ROW = absolute organ weight (g)/body. Mean ± SD (n = 6). * *p* < 0.05 by One-Way ANOVA followed by Bonferroni test vs. control group, ^a^ vs. 5-FU, ^b^ vs. CbPt, ^c^ vs. 6SA and ^d^ vs. AA.

## Data Availability

The data presented in this study are available on request from the corresponding author.

## Supplementary Materials

The following are available online. Figure S1: AA Characterization.
