# Supplementary material for: Anacardic Acids from Amphipterygium adstringens Confer Cytoprotection against 5-Fluorouracil and Carboplatin Induced Blood Cell Toxicity While Increasing Antitumoral Activity and Survival in an Animal Model of Breast Cancer
Source: Molecules. 2021 May 28;26(11):3241. doi: 10.3390/molecules26113241 (PMC8198955; doi:10.3390/molecules26113241)
Supplement: Supplementary file 1 [file molecules-26-03241-s001.zip › molecules-1206546-supplementary.pdf]

IPN
4/12/2019

File: M1-EVD2 **Date Run: 04-12-2019 (Time Run: 12:22:34)**
Sample: M1-EVD2
Instrument: JEOL GCmate
Inlet: Direct Probe **Ionization mode: EI+**

Scan: 769 **R.T.: 10.28**
Base: m/z 304; 4.6%FS TIC: 325408 **#Ions: 201**

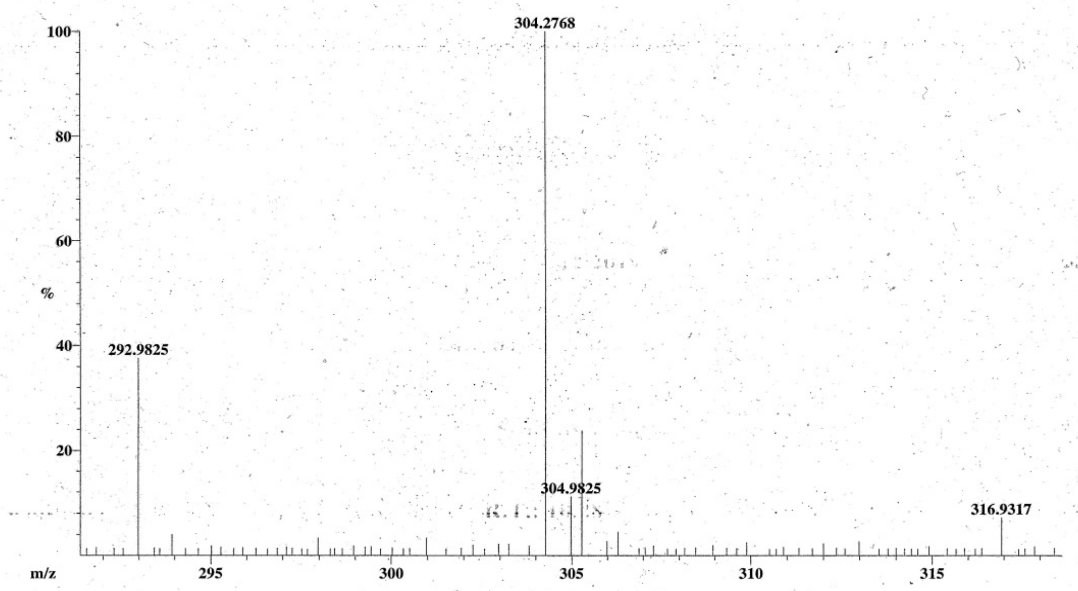

Selected Isotopes : $H_{0\text{-}36}C_{0\text{-}21}O_{0\text{-}1}$ **Error Limit : 5 ppm**

Measured Mass	*% Base*	*Formula*	*Calculated Mass*	*Error*
304.2768	100.0%	$C_{21}H_{36}O$	304.2766	0.6

Supplementary Figure S1. AA characterization. Mass Spectrum (EI^+) of fraction 68. Spectral data. Molecular formula was determined as $C_{22}H_{36}O_3$ by EI^+-MS, the base peak (*m/z*= 304.2768) represents the loss of a carboxyl group.
